# Galectin-1 is associated with the severity of coronary artery disease and adverse cardiovascular events in patients undergoing coronary angiography

**DOI:** 10.1038/s41598-020-77804-6

**Published:** 2020-11-26

**Authors:** Ruey-Hsing Chou, Shao-Sung Huang, Chin-Sung Kuo, Shen-Chih Wang, Yi-Lin Tsai, Ya-Wen Lu, Chun-Chin Chang, Po-Hsun Huang, Shing-Jong Lin

**Affiliations:** 1 Division of Cardiology, Department of Medicine, Taipei Veterans General Hospital, 112, No. 201, Sec. 2, Shih-Pai Road, Taipei, Taiwan; 2 Department of Critical Care Medicine, Taipei Veterans General Hospital, Taipei, Taiwan; 3 Cardiovascular Research Center, National Yang-Ming University, Taipei, Taiwan; 4 Institute of Clinical Medicine, National Yang-Ming University, Taipei, Taiwan; 5 Healthcare and Management Center, Taipei Veterans General Hospital, Taipei, Taiwan; 6 Department of Medical Research, Taipei Veterans General Hospital, Taipei, Taiwan; 7 Division of Endocrinology and Metabolism, Department of Medicine, Taipei Veterans General Hospital, Taipei, Taiwan; 8 Department of Anesthesiology, Taipei Veterans General Hospital, Taipei, Taiwan; 9 School of Medicine, National Yang Ming University, Taipei, Taiwan; 10 Taipei Heart Institute, Taipei Medical University, Taipei, Taiwan; 11 Division of Cardiology, Heart Center, Cheng-Hsin General Hospital, Taipei, Taiwan

**Keywords:** Biomarkers, Cardiology

## Abstract

Galectin-1, a β-galactoside-binding lectin mediating inflammation and neovascularization, is reported to attenuate ventricular remodeling after myocardial infarction. But its role in stable coronary artery disease (CAD) has not been fully elucidated. This study aimed to identify the relationship between the circulating galectin-1 level and the severity of CAD in patients with suspected CAD. Pre-procedure galectin-1 and high-sensitivity C-reactive protein (hs-CRP) concentrations were measured in 834 subjects who underwent scheduled coronary angiography. Subjects were grouped into tertiles of the galectin-1 levels. SYNTAX scores were calculated to evaluate the severity of CAD. All patients were followed until January 2019 or the occurrence of major adverse cardiovascular events (MACE). Patients with higher galectin-1 concentrations were older; had greater prevalence of hypertension, diabetes, chronic kidney disease, and heart failure; and were more likely to present with higher hs-CRP levels and SYNTAX scores. During the follow-up period of 1.3 ± 1.1 years, patients in the highest tertile of galectin-1 were associated with a greater risk of MACE after adjustment for age, sex, comorbidities, co-medications, serum levels of hemoglobin, creatinine, hs-CRP, ejection fraction, SYNTAX scores, and revascularization modalities (adjusted hazard ratio 10.95, 95% confidence interval 2.29–52.47, *p* = 0.003). Galectin-1 showed better discriminatory performance than hs-CRP, and non-inferior performance to SYNTAX scores, in predicting the incidence of MACE.

## Introduction

Galectins form a group of proteins that can bind to β-galactoside sugars by N-linked or O-linked glycosylation through their carbohydrate recognition domains^1^. Lectin–glycan interactions are involved in numerous physiological and pathological processes, including the regulation of immunity, inflammation, wound healing, and angiogenesis^2,3^. Galectin-1, the first galectin identified, consists of two subunits of 14.5 kDa (135 amino acids) and is localized on cell surfaces and in cytoplasm. Galectin-1 may act extracellularly by cross-linking glycoconjugates on cell surfaces, or intracellularly by influencing a variety of signaling pathways^1^. Its main physiological function is as an anti-inflammatory mediator, repressing the innate and adaptive immune response^4^. Recent evidence indicates that galectin-1 plays an essential role in cardiovascular pathophysiology by moderating acute and chronic inflammatory responses^5^. Increased galectin-1 expression has been found in the cardiomyocytes of patients with myocardial infarction (MI), heart failure, and Chagas cardiomyopathy^6^. Galectin-1-deficient mice have been reported to show enhanced cardiac inflammation and worse ventricular remodeling after acute MI^7^. Nevertheless, evidence for the role of galectin-1 in chronic coronary artery disease (CAD) is very limited. Although animal and cell line studies have been conducted, no clinical study has demonstrated the relationship between galectin-1 and CAD.

In this single-center observational study, we aimed to investigate the relationships of the serum galectin-1 concentration to the severity of CAD and the occurrence of subsequent cardiovascular events in patients undergoing elective coronary angiography. We hypothesized that increasing pre-procedure serum galectin-1 concentrations would be associated with more severe CAD and worse clinical outcomes in patients with suspected CAD. To explore the role of galectin-1 in inflammation, we also measured the pre-procedure serum concentrations of high-sensitivity C-reactive protein (hs-CRP), a widely used inflammatory biomarker that is known to predict cardiovascular outcomes^8^. To validate the diagnostic and prognostic values of galectin-1, we compared its predictive power with that of hs-CRP.

## Methods

### Patient enrollment and galectin-1 measurement

**Figure 1:**
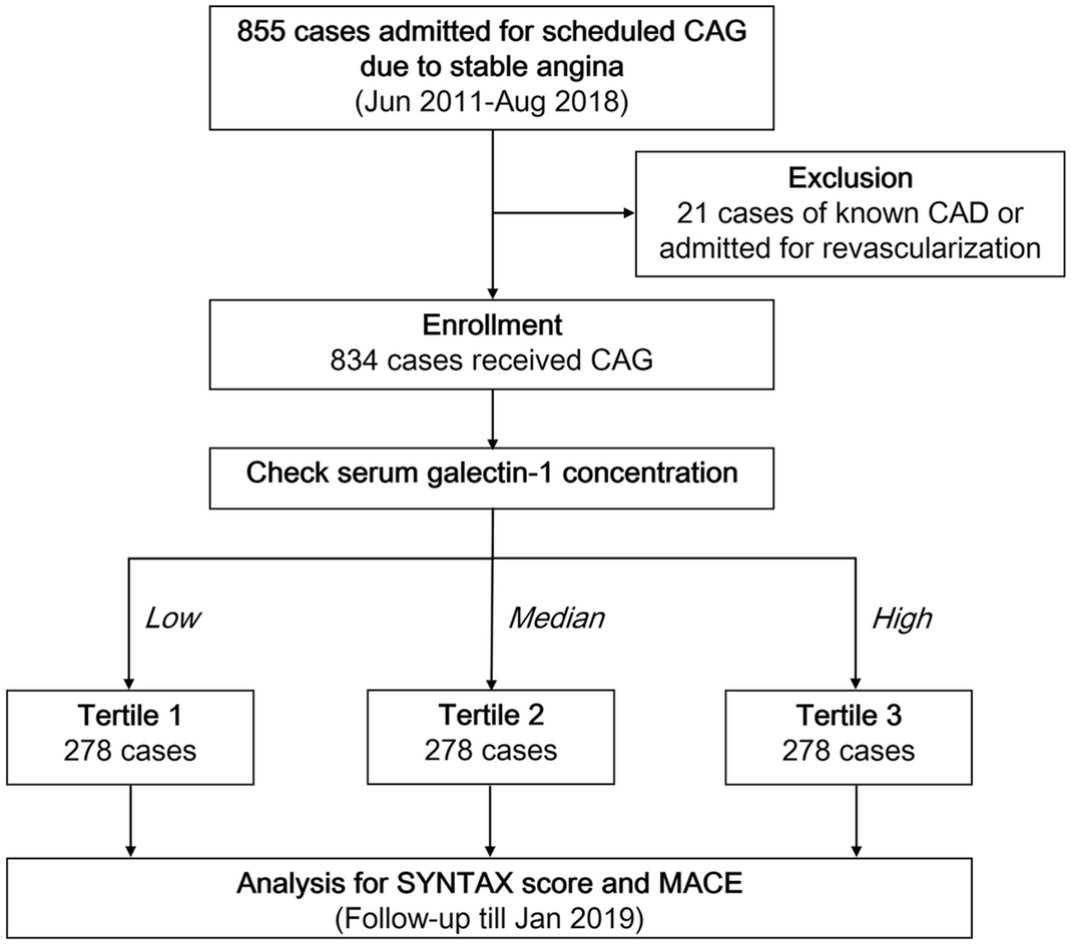
Flowchart of patient enrollment and follow-up. CAD, coronary artery disease; CAG, coronary angiography; MACE, major adverse cardiovascular events.

From January 2011 to August 2018, we retrospectively screened 855 patients aged > 18 years who were admitted for scheduled cardiac catheterization due to stable angina and suspected CAD in the Taipei Veterans General Hospital. After excluding 21 cases with documented CAD or admitted for revascularization, we enrolled 834 patients in this study. Information about patients’ age, sex, body mass index (BMI), smoking history, comorbidities, and co-medications was collected via detailed chart review. Pre-procedure blood samples were obtained after at least 8 h of fasting. The hemoglobin and serum levels of glucose, creatinine, cholesterol, and triglycerides were determined using routine laboratory methods with a Hitachi 7600 autoanalyzer (Hitachi Ltd., Tokyo, Japan). Serum hs-CRP levels were determined using a commercial enzyme-linked immunosorbent assay (Beckman Coulter Inc., Brea, CA, USA). Serum galectin-1 concentrations were also determined by commercial enzyme-linked immunosorbent assay (R&D Systems, Inc., Minneapolis, MN, USA); the sensitivity was 0.129 ng/mL and the assay range was 0.3–20 ng/mL. Intra- and inter-assay coefficients were 5.7–8.8% and 7.5–9.5%, respectively. All patients were grouped into tertiles [*n* = 278 (33.3%) each] according to their baseline serum galectin-1 concentrations (low, galectin-1 level < 16.1 ng/mL; median, galectin-1 level ≥ 16.1 ng/mL and ≤ 22.7 ng/ml; high, galectin-1 level > 22.7 ng/mL). A flowchart of patient enrollment and grouping is provided as Fig. 1. This research was conducted according to the principles expressed in the Declaration of Helsinki. All participants provided written informed consent, and the study was approved by the Research Ethics Committee of Taipei Veterans General Hospital.

### Severity of CAD and study endpoints

Two experienced interventional cardiologists interpreted all coronary angiograms. SYNTAX scores were calculated to estimate the severity of CAD^9^. Each coronary lesion with ≥ 50% stenosis in a vessel with a diameter > 1.5 mm was scored. The study cohort was further divided into tertiles of SYNTAX score (0, 1–22, and > 22) for the evaluation of prognosis^10^. Additionally, left ventriculography was performed to estimate left ventricular ejection fractions (LVEFs).

Modalities of revascularization, including percutaneous coronary intervention (PCI) and coronary artery bypass grafting (CABG), were obtained by chart review. Prescriptions of medications after CAG, including aspirin, clopidogrel, angiotensin-converting enzyme inhibitor (ACEi)/angiotensin receptor blocker (ARB), beta-blocker, and statin, were also recorded. All patients were advised to visit outpatient clinics 1 week after the procedure, then every 3 months for a refill of medications. Their medical records were reviewed regularly until the occurrence of major adverse cardiovascular events (MACE), including target vessel revascularization, non-fatal MI, non-fatal stroke, and death. Target vessel revascularization was defined as balloon dilatation or stent deployment over a previously treated lesion. Non-fatal MI was defined as the elevation of cardiac troponins with ischemic symptoms. Non-fatal stroke was defined as the presence of a new neurological deficit with evidence of cerebral infarction, verified by imaging analysis. Detailed definitions of MACE have been provided in our previous work^11^. All study subjects were followed until January 2019. No subject dropped out of the study.

### Statistical analysis

Clinical and laboratory data were compared using the Kruskal–Wallis test for continuous variables (expressed as medians and interquartile ranges) and Chi-squared test for categorical variables (expressed as counts and percentages). Spearman's rank correlation test was used to assess correlations between galectin-1 levels and hs-CRP levels, SYNTAX scores, and other variables. The incidence of MACE was calculated, and survival curves were generated using the Kaplan–Meier method. Survival was compared among patients with different galectin-1 levels, hs-CRP concentrations, and SYNTAX scores using the log-rank test. Cox proportional-hazard regression analysis was performed to identify risk factors for MACE. To assess the independent ability of the galectin-1 level to predict MACE, age, sex and factors that were significant in univariable regression analysis were adjusted for in the multivariable regression analysis. Areas under receiver operating characteristic (ROC) curves (AUCs) were used to evaluate the accuracy of galectin-1, hs-CRP, and SYNTAX scores in predicting the incidence of MACE. Pairwise comparison between two AUCs was performed using the method of DeLong et al^12^. Data were analyzed using SPSS version 18.0 (SPSS Inc., Chicago, IL, USA) and MedCalc version 11.4.2.0 (MedCalc Software, Mariakerke, Belgium). *p* values < 0.05 were regarded as significant.

## Results

### Baseline characteristics

In total, 834 patients who underwent elective coronary angiography were enrolled for analysis. The mean age of the study population was 66.8 ± 12.3 years, and 68.2% of the patients were male. The clinical and demographic characteristics of the patients are summarized in Table 1. Patients in the low and high galectin-1 groups did not differ with respect to sex, smoking status, or serum level of fasting glucose or total cholesterol. However, compared with patients in the lowest galectin-1 tertile, those with higher serum galectin-1 concentrations were older; had greater prevalence of hypertension, diabetes, and chronic kidney disease; with higher percentages of using aspirin, clopidogrel, ACEi/ARB, beta-blocker; and were more likely to present with higher levels of serum creatinine, triglycerides, and hs-CRP, higher BMI and SYNTAX scores. Individuals with higher galectin-1 levels also had greater prevalence of heart failure, peripheral artery disease, and prior history of stroke. Galectin-1 and hs-CRP concentrations correlated positively with age, BMI, WBC count, fasting glucose level, and SYNTAX score, and were associated negatively with the hemoglobin concentration and LVEF. The SYNTAX score also correlated significantly with age, LVEF, and the hemoglobin, fasting glucose, creatinine, and total cholesterol levels. Correlations between the circulating galectin-1 level, hs-CRP level, SYNTAX score, and various clinical variables are summarized in Supplemental Table 1.

**Table 1:** Baseline characteristics of the study cohort by tertiles of serum galectin-1 concentrations.

Characteristic	Tertile 1 (n=278) Galectin 1 < 16.1	Tertile 2 (n=278) Galectin 1: 16.1–22.7	Tertile 3 (n=278) Galectin 1 > 22.7	*p*
Age (years)	61.0 (54.8–71.0)	67.0 (60.0–75.0)	72.0 (61.0–80.3)	< 0.001
Male, n (%)	188 (67.6)	184 (66.2)	197 (70.9)	0.479
Smoking, n (%)	79 (28.4)	99 (35.6)	99 (35.6)	0.115
Body mass index (kg/m2)	24.7 (22.5–27.6)	25.9 (23.9–28.5)	25.3 (23.0–28.0)	0.005
**Comorbidities**				
Hypertension	153 (55.0)	185 (66.5)	219 (78.8)	< 0.001
Diabetes	67 (24.1)	90 (32.4)	123 (44.2)	< 0.001
Chronic kidney disease	5 (1.8)	7 (2.5)	71 (25.5)	< 0.001
Heart failure	10 (3.6)	8 (2.9)	44 (15.8)	< 0.001
Peripheral arterial disease	21 (7.6)	12 (4.3)	39 (14.0)	< 0.001
Previous stroke	14 (5.1)	12 (4.3)	25 (9.0)	0.047
**Co-medications**				
Aspirin	129 (46.4)	120 (43.2)	151 (54.3)	0.026
Clopidogrel	34 (12.2)	39 (14.0)	60 (21.6)	0.006
ACEi/ARB	62 (22.3)	85 (30.6)	105 (37.8)	< 0.001
Beta-blocker	54 (19.4)	69 (24.8)	85 (30.6)	0.010
Statin	85 (30.6)	96 (34.5)	83 (29.9)	0.443
**Laboratory data**				
Hemoglobin (g/dL)	13.5 (12.5–14.3)	13.4 (12.4–14.3)	12.3 (10.8–13.7)	< 0.001
Fasting glucose (mg/dL)	98.0 (87.3–119.0)	98.0 (89.0–114.0)	102.0 (89.0–130.8)	0.089
Creatinine (mg/dL)	0.9 (0.8–1.1)	1.0 (0.9–1.2)	1.3 (1.1–1.9)	< 0.001
Total cholesterol (mg/dL)	160.0 (143.0–182.0)	159.0 (137.0–181.5)	158.0 (136.5–182.0)	0.328
Triglycerides (mg/dL)	99.0 (71.0–138.5)	107.0 (77.0–148.8)	112.0 (86.5–160.0)	0.001
Hs-CRP (mg/dL)	0.1 (0.0–0.2)	0.1 (0.0–0.3)	0.2 (0.1–0.7)	< 0.001
Galectin 1 (ng/mL)	13.3 (10.2–14.7)	18.7 (17.4–20.5)	29.3 (25.5–38.5)	< 0.001
**Cardiac catheterization**				
Single vessel disease, *n* (%)	55 (19.8)	69 (24.8)	59 (21.2)	0.336
Multiple vessel disease, *n* (%)	69 (24.8)	80 (28.8)	120 (43.2)	< 0.001
SYNTAX score	0.0 (0.0–5.0)	0.0 (0.0–7.0)	7.0 (0.0–16.1)	< 0.001
LV ejection fraction (%)	59.0 (54.6–62.2)	58.0 (52.0–63.0)	55.9 (49.0–60.9)	0.009

ACEi, angiotensin-converting enzyme inhibitor; ARB, angiotensin receptor blocker; CAD, coronary artery disease; Hs-CRP, high sensitive C-reactive protein; LV ejection fraction, left ventricular ejection fraction; MACE, major adverse cardiovascular events.

### Study endpoints and the prognostic value of galectin-1

Totally 452 (54.2%) patients were found to have significant CAD, including 183 (21.9%) cases of single-vessel disease (SVD) and 269 (32.3%) cases of multiple vessel disease (MVD). Four-hundreds and thirty-eight patients (n = 438, 52.5%) underwent revascularization, either by PCI (n = 403, 48.3%) or by CABG (n = 35, 4.2%). Patients with higher galectin-1 concentrations were with higher prevalence of MVD, with higher rate of receiving PCI or CABG, and placed more stents while undergoing PCI (Table 2). After CAG, patients with higher galectin-1 concentrations were also received more prescriptions of aspirin, clopidogrel, ACEi/ARB, and beta-blocker.

**Table 2:** Modalities of revascularization, medications after coronary angiography (CAG), and adverse cardiovascular events based on tertiles of serum galectin-1 levels.

	Galectin-1 Tertiles			*p*
	<16.1 ng/mL	16.1–22.7 ng/mL	>22.7 ng/mL	
	(n = 278)	(n = 278)	(n = 278)	
**Types of revascularization**				
Percutaneous coronary intervention (PCI)	108 (38.8)	138 (49.6)	157 (56.5)	< 0.001
Number of stents	2.0 (1.0–3.0)	2.0 (1.0–3.0)	2.0 (1.0–4.0)	0.001
Coronary artery bypass grafting (CABG)	10 (3.6)	7 (2.5)	18 (6.5)	0.055
Number of grafts	3.0 (3.0–3.0)	3.0 (3.0–4.0)	3.0 (3.0–4.0)	0.155
**Medications after CAG,** ***n*** **(%)**				
Aspirin	179 (64.4)	192 (69.1)	216 (77.7)	0.002
Clopidogrel	118 (42.4)	144 (51.8)	169 (60.8)	< 0.001
ACEi/ARB	77 (27.7)	102 (36.7)	132 (47.5)	< 0.001
Beta-blocker	59 (21.2)	75 (27.0)	92 (33.1)	0.007
Statin	120 (43.2)	140 (50.4)	137 (49.3)	0.187
**Adverse cardiovascular events,** ***n*** **(%)**				
Target vessel revascularization	17 (6.1)	13 (4.7)	43 (15.5)	< 0.001
Nonfatal MI	1 (0.4)	1 (0.4)	7 (2.5)	0.018
Nonfatal stroke	0 (0.0)	1 (0.4)	2 (0.7)	0.367
Death	4 (1.4)	1 (0.4)	13 (4.7)	0.001
Overall MACE	22 (7.9)	16 (5.8)	65 (23.4)	< 0.001

Values are number (%). ACEi, angiotensin-converting enzyme inhibitor; ARB, angiotensin receptor blocker; MI, myocardial infarction; MACE, major adverse cardiovascular event.

**Figure 2:**
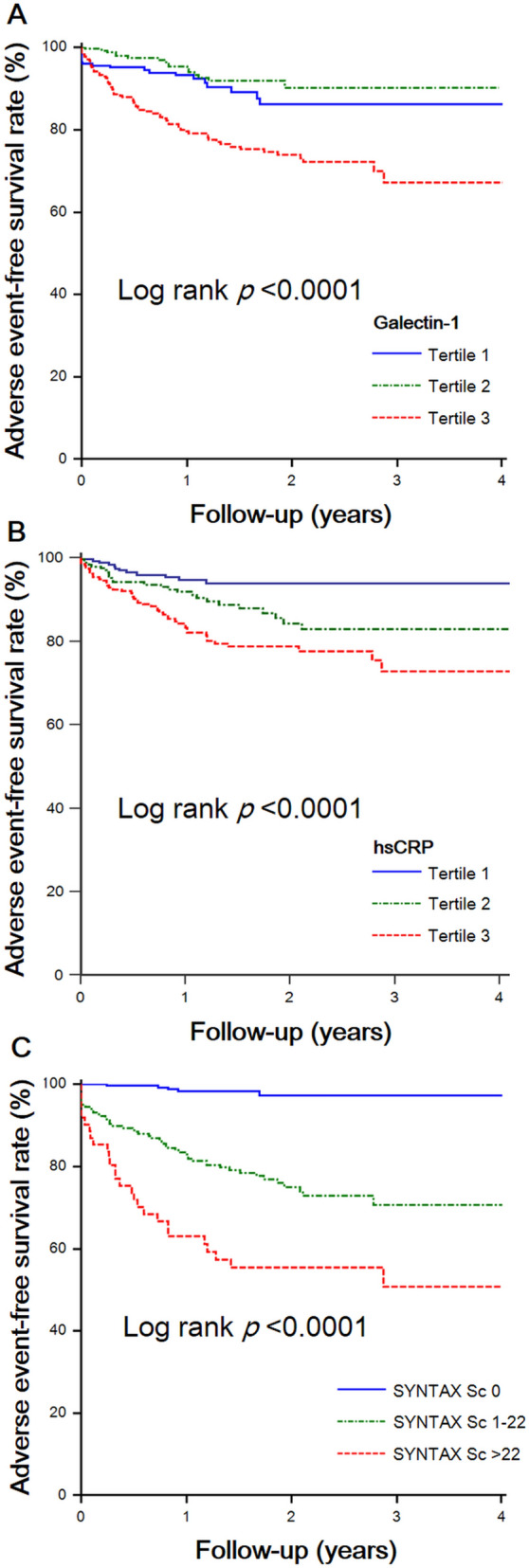
Kaplan–Meier curves of freedom from major adverse cardiac events by tertiles of serum (**A**) galectin-1, (**B**) high-sensitive C-reactive protein (hsCRP) concentration, or by (**C**) difference of SYNTAX scores.

During the follow-up period of 1.3 ± 1.1 years, 103 (12.4%) MACE occurred, consisting of 73 (8.8%) cases of target vessel revascularization, 9 (1.1%) non-fatal MIs, 3 (< 0.1%) non-fatal strokes, and 18 (2.2%) deaths. Patients in the high galectin-1 group exhibited more target vessel revascularization, non-fatal MI, and all-cause mortality than did those in the low galectin-1 group. The incidence of MACE was significantly higher in patients with high galectin-1 levels than in those with low galectin-1 levels (23.4% vs. 7.9%, *p* < 0.001). However, the association between the galectin-1 level and incident ischemic stroke was not significant. Kaplan–Meier survival analysis was performed to investigate the potential impact of baseline galectin-1 levels on adverse event–free survival. Patients in the highest galectin-1 tertile had a significantly lower incidence of MACE-free survival than did patients in the lowest galectin-1 tertile (log-rank *p* < 0.001; Fig. 2). Moreover, patients with higher hs-CRP concentrations or higher SYNTAX scores had significantly lower incidences of MACE-free survival.

Hypertension, diabetes, heart failure, peripheral artery disease, the usage of clopidogrel or ACEi/ARB; serum levels of hemoglobin, creatinine, hs-CRP, and galectin-1; MVD, SYNTAX scores, LVEF, and revascularization with PCI or CABG were all significantly associated with the incidence of MACE in univariable Cox regression analysis (Table 3). Patients in the highest galectin-1 tertile had a significantly greater risk of MACE after adjustment for age, sex, serum hs-CRP concentration, and SYNTAX score [adjusted hazard ratio (aHR) 5.70, 95% confidence interval (CI) 2.24–14.47,* p* < 0.001]. The highest tertile of galectin-1 remained independently associated with the occurrence of MACE after adjustment for age, sex, hypertension, diabetes, heart failure, peripheral artery disease, the usage of clopidogrel or ACEi/ARB, serum levels of hemoglobin, creatinine, hs-CRP, LVEF, SYNTAX score, MVD, and revascularization with PCI or CABG (aHR 10.95, 95% CI 2.29–52.47, *p* = 0.003). In contrast, the association between the serum hs-CRP concentration and MACE became insignificant in the multivariable regression analysis.

**Table 3:** Multivariable Cox proportional hazard analysis for serum galectin-1, hsCRP concentration, and anatomical SYNTAX score (SYNTAX Sc) to the incidence of major adverse cardiovascular events (MACE).

	Univariable		Model 1*		Model 2✝	
Variables	Crude HR (95% CI)	*P*	Adjusted HR (95% CI)	*P*	Adjusted HR (95% CI)	*p*
**Galectin-1**						
*tertile 1*	*Reference*		*Reference*		*Reference*	
*tertile 2*	0.63 (0.33–1.20)	0.161	1.79 (0.60–5.36)	0.297	4.42 (0.76–25.81)	0.099
*tertile 3*	2.35 (1.45–3.82)	0.001	5.70 (2.24–14.47)	<0.001	10.95 (2.29–52.47)	0.003
Age	1.01 (1.00–1.03)	0.115	1.01 (0.99–1.03)	0.320	1.01 (0.98–1.05)	0.415
Gender	1.47 (0.94–2.31)	0.094	1.10 (0.59–2.06)	0.755	0.79 (0.33–1.87)	0.593
BMI	0.98 (0.94–1.03)	0.451				
HTN	2.60 (1.54–4.37)	< 0.001			1.30 (0.44–3.86)	0.633
Diabetes	1.89 (1.28–2.78)	0.001			1.77 (0.87–3.58)	0.113
HF	2.81 (1.69–4.68)	< 0.001			1.70 (0.72–4.01)	0.224
PAD	2.34 (1.42–3.85)	0.001			2.13 (0.71–6.33)	0.176
Stroke	1.52 (0.77–3.00)	0.234				
Aspirin	1.41 (0.95–2.08)	0.085				
Clopidogrel	1.78 (1.13–2.79)	0.013			0.52 (0.22–1.26)	0.148
ACEi / ARB	1.99 (1.35–2.94)	0.001			0.89 (0.44–1.80)	0.747
BB	1.26 (0.82–1.95)	0.292				
Hb	0.81 (0.73–0.89)	< 0.001			1.26 (1.04–1.54)	0.021
Creatinine	1.18 (1.12–1.25)	< 0.001			1.15 (1.01-1.30)	0.035
TC	1.00 (0.99–1.00)	0.094				
TG	1.00 (1.00–1.00)	0.053				
hsCRP	1.12 (1.06–1.17)	< 0.001	1.08 (0.99–1.18)	0.099	1.12 (0.91–1.37)	0.293
MVD	4.55 (3.01–6.86)	< 0.001			1.49 (0.54–4.12)	0.443
SYNTAX Sc	1.06 (1.05–1.08)	< 0.001	1.05 (1.04–1.07)	<0.001	0.99 (0.94–1.04)	0.678
LVEF	0.97 (0.95–0.99)	0.003			1.00 (0.97–1.03)	0.942
Received PCI	3.38 (2.16–5.31)	< 0.001			11.80 (2.48–56.07)	0.002
Received CABG	2.72 (1.45–5.08)	0.002			10.93 (0.88–137.59)	0.064

* = adjusted for age, gender, hs-CRP, and SYNAX scores.

✝ = adjusted for age, gender, and variables with *p* value < 0.05 in the univariable analysis.

ACEi, angiotensin-converting enzyme inhibitor; ARB, angiotensin receptor blocker; BB, beta-blocker; BMI, body mass index; CABG, coronary artery bypass grafting; Hb, hemoglobin; HDL, high-density lipoprotein; HF, heart failure; HTN, hypertension; hs-CRP, high sensitivity C-reactive protein; LVEF, left ventricular ejection fraction; MVD, multiple vessel disease; PAD, peripheral arterial disease; PCI, percutaneous coronary intervention; TC, total cholesterol; TG, triglycerides.

### Predictive power of galectin-1

**Figure 3:**
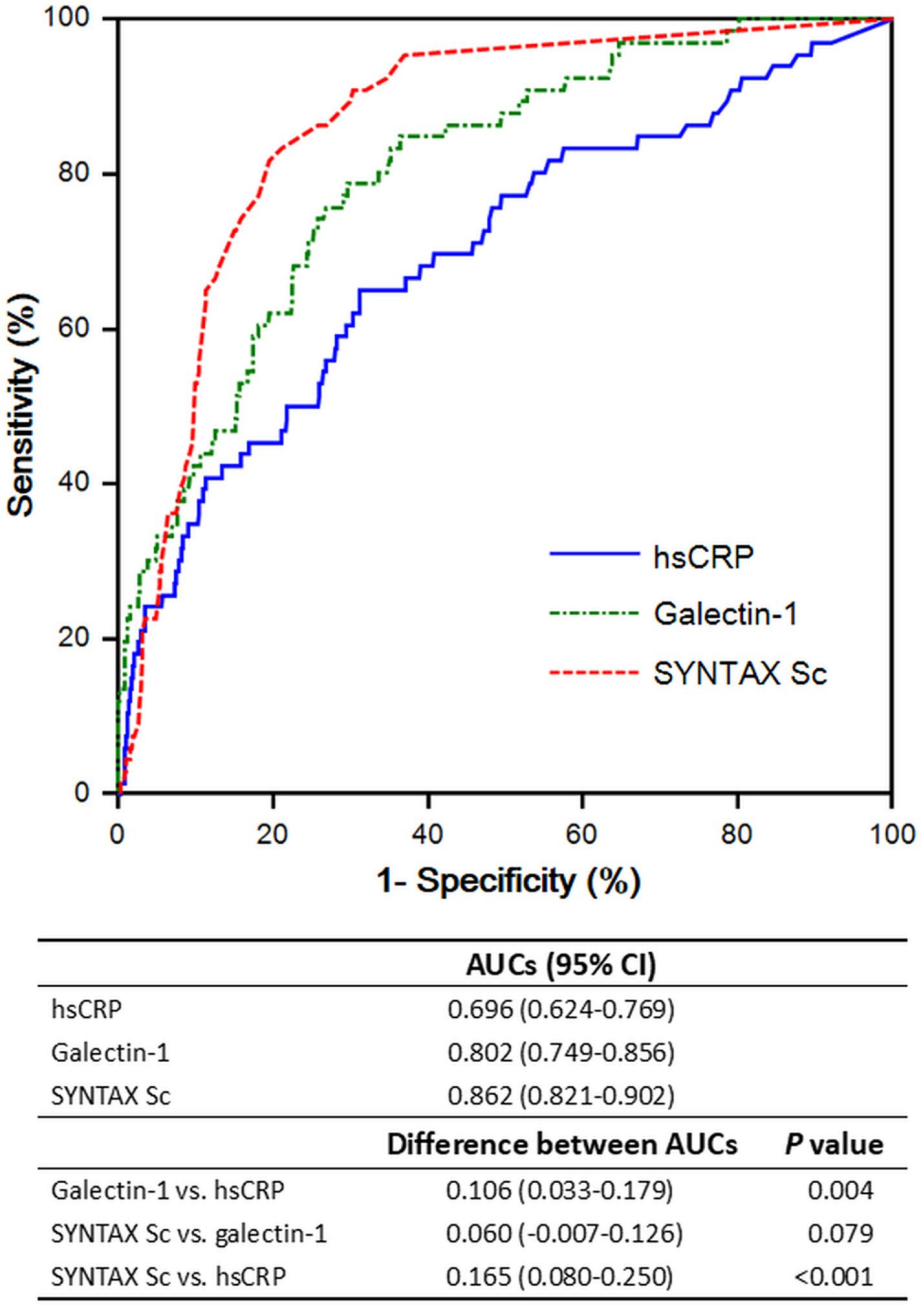
C-statistics and pairwise comparison of the ROC curves between serums C-reactive protein (hsCRP), galectin-1, and SYNTAX score (SYNTAX Sc) in prediction the incidence of major adverse cardiovascular events (MACE). Comparisons between 2 AUCs were performed using the method of DeLong et al. AUC, area under the curve of ROC; ROC curve, receiver operating characteristic curve.

ROC curves characterizing the ability of the serum galectin-1 level, hs-CRP concentration, and SYNTAX score to predict the incidence of MACE are presented in Fig. 3. The SYNTAX score had the greatest AUC (0.862), followed by the serum galectin-1 level (0.802) and hs-CRP concentration (0.696). In pairwise comparisons, the SYNTAX score (*p* < 0.001) and serum galectin-1 concentration (*p* = 0.004) showed significantly better discriminatory performance than did the serum hs-CRP concentration. The difference in AUCs between the SYNTAX score and serum galectin-1 concentration was not significant. The serum galectin-1 level was non-inferior to the SYNTAX score for the prediction of MACE incidence in patients with suspected CAD.

## Discussion

In this single-center observational study involving 834 patients who underwent elective coronary angiography, the pre-procedure serum galectin-1 concentration was associated significantly with the severity of CAD and subsequent occurrence of MACE. The serum galectin-1 level was an independent predictor of MACE, even after adjustment for comorbidities and the SYNTAX score. As a biomarker, the predictive power of galectin-1 was superior to that of hs-CRP and non-inferior to the SYNTAX score. To our knowledge, this work was the first longitudinal study to investigate the relationship between the circulating galectin-1 level and the occurrence of MACE in patients with suspected CAD. These findings provide novel evidence of the role of galectin-1 in the pathogenesis of atherosclerosis.

Galectin-1 is an evolutionarily conserved β-galactoside–binding lectin that controls immune cell homeostasis in acute and chronic inflammation^6^. Galectin-1 is expressed prominently on the surfaces of inflammatory macrophages^4^, activated T lymphocytes^13^, and tolerogenic dendritic cells^14^. Through cross-linking of cell surface glycoconjugates, galectin-1 may blunt proinflammatory cytokine synthesis, engage T-cell apoptosis, promote the expansion of T regulatory cells, and deactivate antigen-presenting cells^6^. In intracellular signaling, galectin-1 also contributes to neovascularization by enhancing the vascular endothelial growth factor receptor 2 pathway^15^. Because of its prominent anti-inflammatory and pro-angiogenic activities, galectin-1 is suggested to play a crucial role in cardiovascular pathophysiology^7^.

Galectin-1 is found in the heart tissues^16–18^, and is expressed in the cytosolic compartments of cardiomyocytes with an organized striated pattern similar to the sarcomeric actin^18^. Moreover, galectin-1 is upregulated in inflammatory microenvironments^19^ and is an essential component of the hypoxia-regulated transcriptome^20^. Therefore, galectin-1 has been proposed to be a key mediator of post-MI ventricular remodeling, and of heart failure^7^. In vitro, cardiomyocytes upregulate and secrete galectin-1 soon after hypoxia in response to proinflammatory cytokines^7^. Increased galectin-1 expression in heart tissues was observed in a mouse model of MI^16^ and in patients with advanced heart failure^7^.

Atherosclerosis is a complex process with multifactorial etiologies, including abnormal lipid metabolism, inflammation, endothelial dysfunction, fibroproliferation, and angiogenesis. The progression of atherosclerotic plaque can lead to several cardiovascular diseases, such as CAD, MI, heart failure, and stroke, which remain the major causes of death worldwide. Inflammation plays a vital role in the development of atherosclerosis^21^; it can influence lipoprotein transfer to the vessel wall. Proinflammatory cytokines, such as tumor necrosis factor-α, increase the binding of low-density lipoprotein (LDL) cholesterol to the endothelium^22^. Inflammation also stimulates leukocytes to release reactive oxygen species and oxidize LDL. Macrophages engulfing oxidized LDL transform into foam cells, which are deposited in the subendothelial space, forming the fatty streak of atherosclerotic plaque^23^. As galectin-1 can modulate the inflammatory response in cardiomyocytes, galectin-dependent signaling is an attractive target for the diagnosis and treatment of cardiovascular disease.

The exact mechanism linking galectin-1 to CAD remains unclear. Previous reports have implicated a possible pathway between galectin-1 and MI^7,16^. In an animal study of MI induced by coronary artery ligation, increased galectin-1 expression was identified in the cardiomyocytes of infarct areas 7 days after tissue ischemia^7^. Compared with their wild-type counterparts, mice lacking galectin-1 (*Lgals1*^*−/*−^) were found to have enhanced cardiac inflammation, attenuated heart function, and dilated heart chambers after non-reperfused MI^7^. In in vitro studies^24,25^, galectin-1 promoted apoptosis of Th1 and Th17 lymphocytes, which is associated with myocardial injury and adverse remodeling after MI^26^, and results in a shift toward a Th2-dominant cytokine^24^. Furthermore, treatment with recombinant galectin-1 mitigated the cardiac damage of MI in *Lgals1*^*−/−*^ mice^7^.

Several pathways may explain the associations between galectin-1 elevation and the incidence of MACE observed in our study. As galectin-1 has protective effects against acute MI, elevated serum galectin-1 levels may reflect the compensation of chronic vascular inflammation in patients with CAD. However, in contrast to the robust evidence for MI, evidence suggesting that galectin-1 attenuates inflammation in stable CAD is very limited and controversial. ApoE^−/−^ mice fed a high-cholesterol diet showed increased galectin-1 expression in atherosclerotic plaques, but the expression did not increase further over time^27^. In addition, statin therapy inhibited the expression of galectin-3, but did not affect galectin-1^27^. Another possible explanation is that galectin-1 is an early-stage byproduct of atherosclerosis. Galectin-1 was reported to be involved in the adhesion and proliferation of vascular smooth muscle cells (SMCs)^28^. Galectin-1 messenger RNA levels are significantly increased in growing SMCs compared with resting SMCs^29^. Instead of a potent antioxidant acting against vascular inflammation, galectin-1 is more likely to be a mediator involved in the development of atherosclerosis in chronic CAD.

Although the association between galectin-1 and stroke has been established^30^, no clinical study has investigated the role of galectin-1 in patients with CAD. Our study findings suggest that progressive increases in the serum galectin-1 concentration are associated with the severity and prognosis of CAD. In the present study, the circulating galectin-1 level correlated with the WBC count and serum hs-CRP level, reflecting the physiological role of galectin-1 in modulating inflammation. Furthermore, the predictive power of galectin-1 for MACE was superior to that of hs-CRP. As an acute-phase protein synthesized in the liver, hs-CRP is elevated in CAD and all inflammation-associated diseases^21^. Compared with systemic inflammation, galectin-1 elevation may proceed via a different mechanism in myocardial injury, making it more specific to cardiovascular disease. In a mouse model of MI, galectin-1 expression was found to peak as early as 20 min after coronary artery ligation^16^, followed by another peak on day 7^7^. This bimodal phenomenon may reflect the expression of galectin-1 by different cells after MI: ischemic cardiomyocytes in the early phase and leukocytes to attenuate inflammation in the delayed phase. Our findings, in agreement with previous reports, support the role of galectin-1 in vascular inflammation and atherosclerosis in patients with CAD.

Several limitations of this study should be addressed. First, the study population was relatively small and was recruited from a single center. Further research with larger samples is required to confirm our findings. Second, the patients enrolled in our study were older (mean age 66.8 ± 12.3 years) and had a high prevalence of comorbidities. Caution should be taken while applying our results to younger populations. Finally, due to the retrospective nature of this study, we could not investigate changes in the galectin-1 concentration in the follow-up period. The discovery that the serum galectin-1 level is continuously elevated in patients who experience MACE would have a greater clinical impact.

In conclusion, the circulating galectin-1 level was associated with the severity of CAD and subsequent occurrence of MACE in patients with suspected CAD. The predictive power of galectin-1 was superior to that of hs-CRP and non-inferior to that of the SYNTAX score. These findings provide novel evidence of galectin-1′s involvement in vascular inflammation and suggest that galectin-1 is an independent prognostic marker of CAD.

## Supplementary information

Supplementary information.
