# Supplementary material for: Galectin-1 is associated with the severity of coronary artery disease and adverse cardiovascular events in patients undergoing coronary angiography
Source: Sci Rep. 2020 Nov 26;10:20683. doi: 10.1038/s41598-020-77804-6 (PMC7692553; doi:10.1038/s41598-020-77804-6)
Supplement: Supplementary file 1 — Supplementary information. [file 41598_2020_77804_MOESM1_ESM.pdf]

SUPPLEMENT

# Galectin-1 is Associated with the Severity of Coronary Artery Disease and Adverse Cardiovascular Events in Patients Undergoing Coronary Angiography

**Ruey-Hsing Chou[1,2,3,4], Shao-Sung Huang[1,4,5,*], Chin-Sung Kuo[4,6], Shen-Chih Wang[7,8], Yi-Lin Tsai[1,3], Ya-Wen Lu[1,3], Chun-Chin Chang[1,3,4], Po-Hsun Huang[1,2,3,4,*], Shing-Jong Lin[1,3,4]**

[1] Division of Cardiology, Department of Medicine, Taipei Veterans General Hospital, Taipei, Taiwan

[2] Department of Critical Care Medicine, Taipei Veterans General Hospital, Taipei, Taiwan

[3] Cardiovascular Research Center, National Yang-Ming University, Taipei, Taiwan

[4] Institute of Clinical Medicine, National Yang-Ming University, Taipei, Taiwan

[5] Healthcare and Management Center, Taipei Veterans General Hospital, Taipei, Taiwan

[6] Division of Endocrinology and Metabolism, Department of Medicine, Taipei Veterans General Hospital, Taipei, Taiwan

[7] Department of Anesthesiology, Taipei Veterans General Hospital, Taipei, Taiwan

[8] School of Medicine, National Yang Ming University, Taipei, Taiwan

**Supplement table 1.** Correlation coefficients of circulating galectin-1, hsCRP concentration, anatomical SYNTAX score (SYNTAX Sc), and various clinical variables

| | **Galectin-1** | | **hsCRP** | | **SYNTAX Sc** | |
|---|---|---|---|---|---|---|
| Variables | **R** | ***P* value** | **R** | ***P* value** | **R** | ***P* value** |
| Age (years) | 0.252 | <0.001 | 0.130 | <0.001 | 0.266 | <0.001 |
| Body mass index (kg/m2) | 0.056 | 0.018 | 0.102 | 0.004 | -0.016 | 0.688 |
| White blood cells (K/cumm) | 0.112 | <0.001 | 0.190 | <0.001 | 0.109 | 0.006 |
| Hemoglobin (g/dL) | -0.229 | <0.001 | -0.181 | <0.001 | -0.185 | <0.001 |
| Fasting glucose (mg/dL) | 0.077 | 0.032 | 0.077 | 0.036 | 0.208 | <0.001 |
| Creatinine (mg/dL) | 0.496 | <0.001 | 0.198 | <0.001 | 0.270 | <0.001 |
| Total cholesterol (mg/dL) | -0.071 | 0.042 | -0.042 | 0.242 | -0.145 | <0.001 |
| Triglycerides (mg/dL) | 0.144 | <0.001 | 0.089 | 0.013 | 0.058 | 0.155 |
| Hs-CRP (mg/dL) | 0.293 | <0.001 | -- | -- | 0.234 | <0.001 |
| SYNTAX Sc | 0.275 | <0.001 | 0.234 | <0.001 | -- | -- |
| LV ejection fraction (%) | -0.160 | 0.004 | -0.158 | 0.005 | -0.250 | <0.001 |

Hs-CRP, high sensitive C-reactive protein; LV ejection fraction, left ventricular ejection fraction.
